# Experimental evidence for short-pulse laser heating of solid-density target to high bulk temperatures

**DOI:** 10.1038/s41598-017-11675-2

**Published:** 2017-09-22

**Authors:** A. Soloviev, K. Burdonov, S. N. Chen, A. Eremeev, A. Korzhimanov, G. V. Pokrovskiy, T. A. Pikuz, G. Revet, A. Sladkov, V. Ginzburg, E. Khazanov, A. Kuzmin, R. Osmanov, I. Shaikin, A. Shaykin, I. Yakovlev, S. Pikuz, M. Starodubtsev, J. Fuchs

**Affiliations:** 1 Institute of Applied Physics of the Russian Academy of Sciences (IAP RAS), 46 Ul’yanov Street, 603950 Nizhny Novgorod, Russia; 2 LULI - CNRS, Ecole Polytechnique, CEA: Université Paris-Saclay; UPMC Univ Paris 06: Sorbonne Universities, F-91128 Palaiseau cedex, France; 3 Joint Institute for High Temperatures Russian Academy of Science (RAS), Moscow, 125412 Russia; 4 PPC and Graduate School of Engineering, Osaka University, 2-1, Yamadaoka, Suita, Osaka, 565-0871 Japan

## Abstract

Heating efficiently solid-density, or even compressed, matter has been a long-sought goal in order to allow investigation of the properties of such state of matter of interest for various domains, e.g. astrophysics. High-power lasers, pinches, and more recently Free-Electron-Lasers (FELs) have been used in this respect. Here we show that by using the high-power, high-contrast “PEARL” laser (Institute of Applied Physics-Russian Academy of Science, Nizhny Novgorod, Russia) delivering 7.5 J in a 60 fs laser pulse, such coupling can be efficiently obtained, resulting in heating of a slab of solid-density Al of 0.8 µm thickness at a temperature of 300 eV, and with minimal density gradients. The characterization of the target heating is achieved combining X-ray spectrometry and measurement of the protons accelerated from the Al slab. The measured heating conditions are consistent with a three-temperatures model that simulates resistive and collisional heating of the bulk induced by the hot electrons. Such effective laser energy deposition is achieved owing to the intrinsic high contrast of the laser which results from the Optical Parametric Chirped Pulse Amplification technology it is based on, allowing to attain high target temperatures in a very compact manner, e.g. in comparison with large-scale FEL facilities.

## Introduction

The investigation of matter in the so-called Warm dense matter (WDM)^1^ regime is of interest both from a fundamental point of view as well as for applications. This domain of partially-ionized matter is located at the confluence of condensed matter physics, plasma physics, and dense liquids. The matter there is characterized by a density close or above that of a solid, and by temperature ≥1 eV, i.e. by a high energy density (>10^5^ joules per cm^3^). This regime is inherently challenging because Coulomb interactions between particles, atomic physics, as well as quantum effects for the electrons, i.e. electron degeneracy, must be considered, on the contrary to the usual assumptions made in low-density plasma physics or condensed-matter physics. In particular, the thermodynamics behavior of matter in this state cannot be derived in terms of small perturbations as is usually done in ideal models which therefore cannot be easily extended in this regime.

Aside from its fundamental interest, the motivation in investigating WDM is that it is a state of matter that is ubiquitous throughout the Universe, as it is present in planet cores and stars, from the main-sequence ones to neutron stellar atmospheres. Its investigation is also of high relevance for optimizing the ignition of inertially confined fusion fuel since in its path to full compression and heating, the fuel crosses the WDM domain^2^.

To investigate WDM states, and allow theoretical models to progress through benchmarking, samples need to be prepared, starting from conventional, room-temperature, solids. Obviously, homogeneous temperatures and densities, i.e. gradient-free, conditions are desired, but even doing so can be held only over inertial confinement times which are small at those temperatures, hence the samples need to be investigated over very short time-scales (ps).

A variety of experimental facilities exist and more are becoming available that can create and diagnose well-controlled WDM conditions. For a long time, intense, short-pulse lasers offered the best prospects to study WMD; they allowed to heat over ultrafast time scales solids^3^ but were limited to extremely thin (nm-scale) foils due to the limited skin depth penetration of laser light into matter. More recently, ultrafast particles (electrons^4^ and ions^5,6^) generated by the same lasers allowed to heat thicker (µm-scale) samples; however investigations underlined the need, when using electron heating, to have an ultra-high temporal contrast for the laser pulse so that conditions close to gradient-free could be maintained, and the difficulties in avoiding pre-heat of the sample by laser-generated X-rays when using ion heating. Now, the advent of Free-Electron-Lasers (FELs) facilities has opened up new perspectives in the generation of WDM, allowing to heat matter deep in the solid and the ensure energy deposition with minimal gradients^7,8^. Complementary, pulsed-power machines offer the prospect of generating very large volumes of WDM^9^ that would aid in progressing in the understanding of WDM properties.

In the present paper, we show that almost isochoric heating to high temperatures (300 eV) of µm-thick solid-density foil can be achieved in a compact and efficient manner using an ultrahigh contrast, high-power, ultra-short duration laser. First, we present the setup of the experiment performed on the “PEARL” laser facility (Institute of Applied Physics-Russian Academy of Science, Nizhny Novgorod, Russia). Then, we present the experimental characterization of the laser-target coupling conditions, using X-ray and accelerated protons diagnostics. Last, we discuss the bulk heating that is achieved in these conditions, before concluding the study.

## Experimental Setup

The experiments were conducted at the “PEARL” laser facility. It is an Optical Parametric Chirped Pulse Amplification (OPCPA) laser system^10^ with wavelength 910 nm and a power up to 0.56 PW^11^. This technology is in marked difference with alternative technologies in the sense that any signal (including parametric fluorescence, or amplified spontaneous emission (ASE)) cannot be triggered before the pump pulse arrives to the nonlinear parametric crystal (in our case, KD*P). Hence, the ASE duration will be strictly limited to the duration of the pump (i.e. overall 2 ns), as verified by us experimentally using a 50 ps resolution fast diode. Since the main pulse is synchronized in the OPCPA stages to coincide with the pump laser, the duration of the ASE before the main pulse is hence of 1 ns.

**Figure 1:**
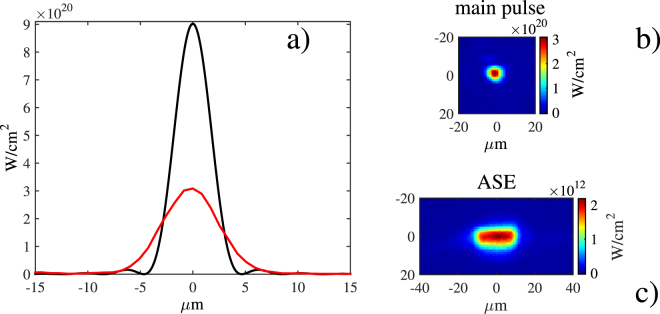
(**a**) Central lineout of the focal spot of the amplified laser (measured at low-flux and here scaled to an energy level of 10 J, red curve) compared to the diffraction-limited spot that would be produced by the laser focusing optics (black curve). (**b**) Measured two-dimensional (2D) focal spot intensity distribution of the amplified laser, from which the lineout shown in (**a**) is taken. (**c**) Same as (**b**) except that the laser is not injected by a coherent seed laser pulse, i.e. this focal spot corresponds to the one of the ASE.

The temporal contrast in intensity, and in the nanosecond range, of the laser system after compression was measured to be 1/(2 × 10^8^)^12^ between the main short pulse and the ASE generated in the OPCPA stages. It is measured as follows: (i) the contrast in energy between the ASE and the main pulse of the compressed laser is equal to 5 × 10^−4^, i.e. 5 mJ compared to 10 J. This is measured by blocking the injection in the chain of the signal pulse that is amplified to lead to the main laser pulse, and by measuring the generated ASE energy. Then, we also (ii) measured that the Strehl ratio of the ASE is 4 times lower than that of the main pulse as shown in Fig. 1. Regarding the picosecond contrast, we can state, as already reported in detail in ref.^12^,that the contrast is of the order of 10^−6^ at 2 ps before the main pulse.

We stress that in the present setup, we did not utilize any contrast enhancement techniques such as plasma mirrors^13^, cross-wave-polarization (XPW)^14^, or double-chirped pulse amplification (CPA)^15^ technique.

**Figure 2:**
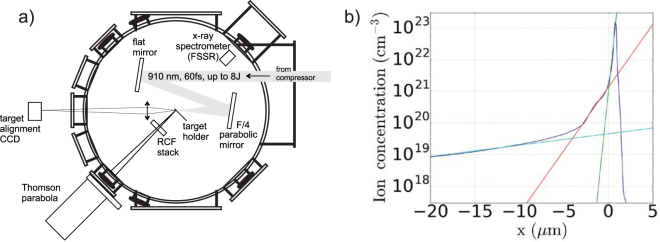
(**a**) Experimental setup; (**b**) two-dimensional (in cylindrical geometry) hydrodynamic simulation of the plasma density profile resulting from the irradiation of a Al 0.8 µm thick target by the amplified spontaneous amplification (ASE) of the short-pulse laser, just prior to the main peak of the laser. Shown is a density lineout along the plasma expansion axis with various fits: (in green) by exp(x/L) with L = 0.16 µm for the overdense part; (in red) by exp(x/L) with L = 1.1 µm for the dense part; and (in cyan) by exp(x/L) with L = 12 µm for the low-density part.

The p-polarized laser pulse originating from the output of the compressor (see Fig. 2a) is focused on the front surface of an Aluminum (Al) foil, having its normal oriented at 45° with respect to the laser axis, by means of a f/4 off-axis parabolic mirror. The on-target laser energy reached 8 J in a 60 fs (full width at half maximum - FWHM) pulse.

Focal spot optimization is performed by means of an adaptive mirror produced by Night N Ltd^16^. For technical and economical reasons, the deformable mirror is located upstream in the laser chain, far from the target (it is placed between the second and the third parametric amplifiers, when the laser is still chirped). The wavefront sensor is located behind the leaky flat transport mirror (see Fig. 2a). The overall wavefront correction system allows to reach a Strehl ratio of 0.36 for the focal spot of 2.9 µm radius (FWHM) with an intensity ~3 × 10^20^ W/cm^2^ for 10 J of laser energy, as illustrated in Fig. 1.

The quality of the focal spot (as shown in Fig. 1) is directly monitored by means of an in-vacuum motorized charge coupled device (CCD)-camera equipped with a microscope objective (not shown in Fig. 2a). The camera completely blocks the fs-laser pulse after focus, and is hence removed from the optical path during the target alignment and for the shot. Al targets are positioned at the location of the best focus; the thickness of these targets was varied from 0.5 µm to 10 µm.

The spectrum of the protons emitted from the rear surface of the target^17,18^, in the so-called target-normal-sheath-acceleration (TNSA) mechanism of ion acceleration from thick solids^19–21^ where ions are electrostatically accelerated by a sheath of hot laser-driven electrons, is measured simultaneously by means of a radiochromic films (RCF) stack^22^ and a Thomson parabola (TP) spectrometer^23,24^, both positioned along the target surface normal, as shown in Fig. 2a. These two diagnostics are complementary: the RCF stack (having a hole in the middle to let a small beam of protons go through toward the Thomson parabola) can analyse the beam in its entirety, but with coarse steps in energy, while the Thomson parabola can resolve finely the spectrum, but only over a small solid angle. The amplitudes of the co-orientated magnetic and electric fields in the Thomson parabola were 0.4 T and 6.5 kV/cm respectively. Its input pinhole of 0.3 mm diameter was situated at a distance of 80 cm from the proton source. The protons in the back of the Thomson parabola were detected using Image Plates (Fujifilm, model TR) (IP)^25–27^ read by a commercial IP-scanner HD-CR 35 NDT procured from DÜRR NDT GmbH & Co. KG. Since we do not have an absolute calibration of the scanner-image plate system, the spectra do not allow us to retrieve the number of accelerated protons. However, this still allows to retrieve accurately the maximal proton energy, as well as to distinguish different types of accelerated ions.

The X-ray emission from the target was measured with the help of a spatially resolved X-ray spectrometer, as will be detailed below.

## Results

### Characterization of the target front surface by X-ray spectrometry

A high-resolution Focusing Spectrometer with Spatial Resolution (FSSR) equipped with a spherically bent mica (K_2_O-3Al_2_O_3_-6SiO_2_-2H_2_O) crystal (2*d* = 19.9376 Å, radius of curvature of *R* = 150 mm) was installed to observe the radiation from the front surface of the target at 30° (in the plane of the laser polarization) to the target surface normal. The crystal was aligned to operate mainly at the *m* = 2 order of reflection to record K-shell emission spectra of multi-charged Al ions in the photon energy range from 1.47 to 1.74 keV. The data were acquired in a time- and space-integrated manner as the detector is passive and as the observed size of X-ray source was well below the diagnostic spatial resolution limit (~50 µm).

**Figure 3:**
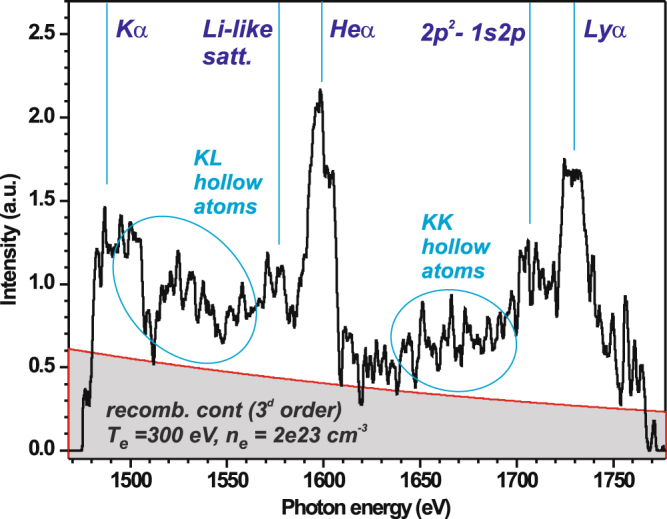
X-ray spectrum measured by means of the FSSR spectrometer from the front surface of an 0.8 µm thick Al target and accumulated in shots #60–64.

To minimize the fluorescence of the crystal due to the impact of fast electrons, a 0.4 T permanent magnet was used that formed a 15 mm wide slit in front of the crystal. Spectra were recorded on “Fujifilm TR” image plates protected against exposure to visible light by a 25 µm thick beryllium window, and were read using the above-mentioned scanner. In order to increase the signal-to-noise rate, the spectrum shown in Fig. 3 was accumulated over four successive shots (# 60–64) performed in similar conditions. It is important to note that all the spectral data is produced following the interaction of the main pulse with the target and that no signal could be recorded as induced solely by the ASE irradiation of the target, i.e. the X-ray emission induced by the sole ASE is completely below the sensitivity of the spectrometer.

The spectrum exhibits Ly_α_ (1727 eV) and He_α_ (1598 eV) resonance lines together with 2p^2^-1s2p satellite and dielectronic satellites to the He_α_ line. The designated position for the neutral K_α_ (1485 eV) line is shown as well, although this line appears unnoticeable. The latter shows that the plasma is multiply ionized even in the vicinity of the laser focal spot.

Apart from the characteristic spectral lines that can observed in Fig. 3, the spectrum consists in a recombination continuum that is reflected by the third order of mica crystal. The recombination continuum, which is consistent with plasma temperatures of ~300 eV in the emitting plasma, becomes dominant in the spectra due to the fact that (i) the reflection coefficient of the 3rd order of mica is several times higher than the one for the 2^nd^ order^28^, as well as (ii) both the transmission of the filter in front of the image plate and the image plate sensitivity^29^ is much higher for harder (2.1–2.5 keV) X-rays.

It is interesting to notice the spectral components that can be observed in two ranges lying between the resonance Ly_α_ and the He_α_ lines, and from the He_α_ dielectronic satellites to the neutral K_α_ line. These spectra components originate from the emission of KK and KL hollow atoms, respectively. In order to describe the full spectra containing both resonance and hollow atom lines, the concept of several plasma zones^30^ with different electronic, radiation temperatures and electronic density should be applied. There, the hot over-ionized central zone irradiated by the laser is considered as a source of intense fast electron flux, which in turn generates ultra-bright X-rays in the keV photon energy range. Hollow atoms can be only created in a solid-density, relatively cold, zone located on the periphery of the central, laser-irradiated zone, by the joint impact of X-rays and fast electrons generated in the central zone. Furthermore, the appearance of KK hollow atoms confirms that (1) the X-ray radiation is intense enough (likely exceeding 10^17^ W/cm^2^) to dominate the kinetics of plasma, as well as that (2) the plasma in the periphery zone remains of solid density^31^. To generate the intense X-rays required for the KK hollow atoms to be produced, the fast electrons produced in the central zone need to be longitudinally refluxed by the sheath fields located at the target surfaces multiple times over, for an efficient electrons-to-X-ray photons energy transfer to take place. For this reflux to take place in an efficient manner, the plasma density gradients at the surfaces need then to be sharp, which suggest that there is no significant plasma expansion in the central zone under the influence of the laser prepulse.

This is consistent with the profile obtained using the hydrodynamic simulation in cylindrical geometry performed with the FLASH code^32^, which is shown in Fig. 2b. We observe that although the prepulse intensity exceeds the ablation threshold and although there is a substantial preplasma extending at low densities (i.e. below 10^20^ cm^−3^) over tens of microns on the target front, most of the target remains at solid-density and the location of the plasma critical density is less than a couple microns away from the initial surface position. As a consequence, the density gradient at the front surface is small and sharp. Quantitatively, we note that, as shown in Fig. 2b with the green and the red curves, that around the critical density, the scalelength L (gauging the local gradient as exp[−x/L]) is in between 0.16 and 1.1 microns. Such gradient scalelength is close to the one reported in ref.^33^ which is, the best of our knowledge, the sharpest gradient (i.e. L ~ 0.6 µm) reported in high-intensity laser-solid interactions that do not resort to plasma mirrors. This is obtained using the PHELIX laser facility at GSI, and this is due to the use, as in our case, of OPCPA technology in order to generate minimum ASE prior to the main short pulse.

We finally note that the observed spectrum containing hollow ion emission contribution differs remarkably from the spectra usually obtained at lower laser intensities (i.e. 10^17^–10^18^ W/cm^2^) containing only resonance lines of H-, He-like Al ions and their satellites (see for example ref.^34^).

Overall, the X-ray spectrum therefore provides a clear evidence that the bulk of the target remains at solid density by the time the main laser pulse arrives, consistently with the hydrodynamic simulation shown in Fig. 2b. Indeed, although the diagnostic is not capable of temporal resolution, we diagnose that the X-ray emission induced by the intense laser pulse irradiation is void of the signature of a significant preplasma at the target front, consistently with what can be derived from Fig. 2b.

### Characterization of the target rear side using accelerated protons measurements

**Figure 4:**
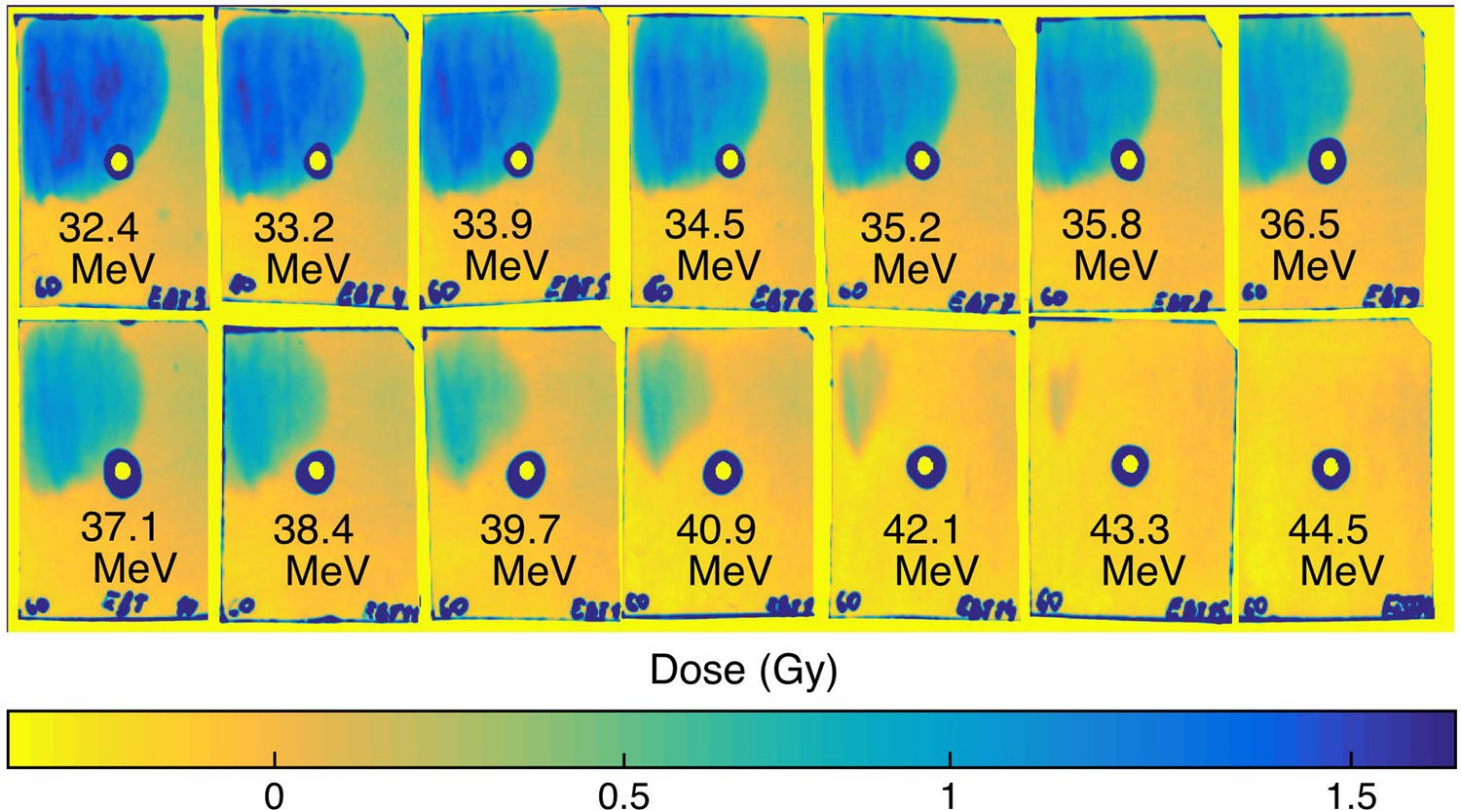
Radiochromic films exposed by the proton beam accelerated by a 7 J laser pulse from an Al 0.8 µm thick target. The energies indicated in each film correspond to the energy of a proton having its Bragg peak in this particular film.

The evidence for small gradients (i.e. L < 1 µm) and solid-density bulk conditions at the target front provided by the X-ray spectrometer is complemented by evidence of similar conditions at the target rear provided by characterizing the ions accelerated from that location. Figure 4 shows the distribution of protons accelerated from the rear surface as measured using RCF films combined into a stack with intermediate aluminum filters. The composition of the stack was Al_1.425mm | HDv2(1) | EBT3(1) | Al_1.425mm | HDv2(1) | EBT3(2) | EBT3(3) | EBT3(4) |… | EBT3(10) | Al_216μm | EBT3(11) | Al_216μm | EBT3(12) |… | Al_216μm | EBT3(20). The stack was composed of two different types of RCF – HDv2 and EBT3^35^ in order to extend its dose dynamic range. The distance from the proton source to the front of the stack was approximately 3.5 cm.

**Figure 5:**
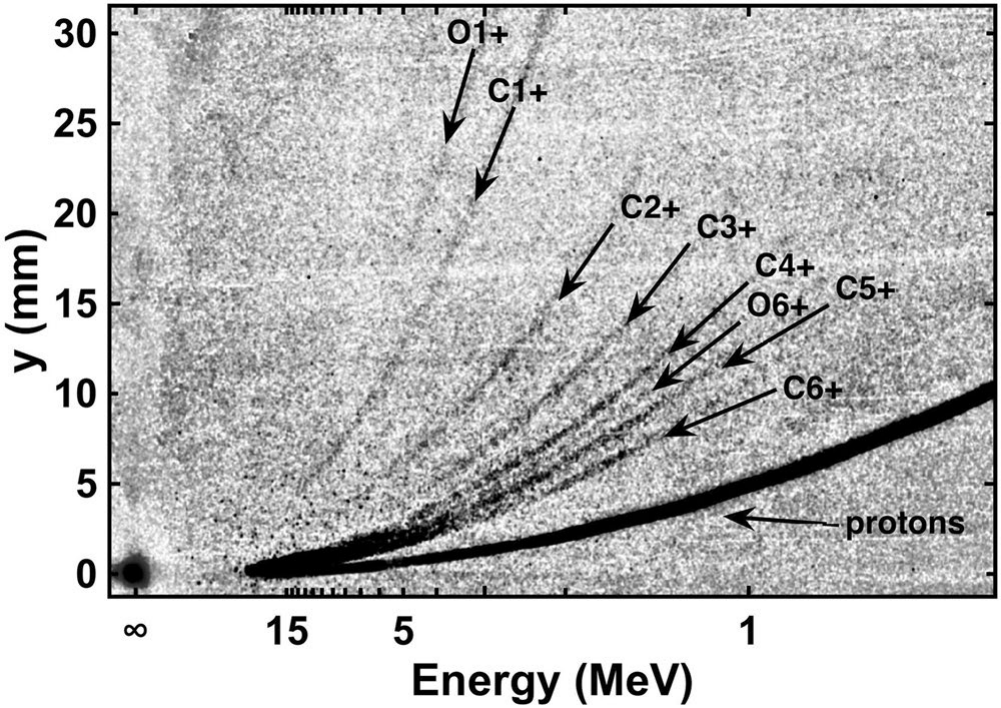
Measurement of the emitted ion spectra as obtained using the Thomson parabola (from an Al 0.8 µm thick target). Traces of H^+^, C^1+^– C^6+^, O^1+^ and O^6+^ ions are marked according to the calculations of the ions’ trajectories. On the horizontal axis is shown the energy of the protons.

Using the Thomson parabola diagnostic, as shown in Fig. 5, we verified that the predominant type of ion, as seen commonly in TNSA^19^, is protons. We observe traces of carbon ions having charges from ranging from C^1+^ to C^6+^ and weak traces of heavier ions that correspond to O^6+^ and O^1+^ according to their charge to mass ratio, but all of these are much weaker than the proton trace. These heavy ions originate from the unavoidable water and organic contamination of the target surface with C-rich molecules^18,36^. Note that the maximal electric field we could deliver to the TP is not enough to distinguish at high energy the various types of the ions from each other because the traces of heavy ions overlap with the proton trace above 15 MeV. To solve this problem, we used two IPs stacked on each other. The second IP displayed only a trace corresponding to the energetic part of the proton spectrum, since the heavier ions and slow protons were blocked by the first IP. Such use of a second IP allowed us to determine the maximum proton energy more accurately, which is well-consistent with what is retrieved from the RCF.

**Figure 6:**
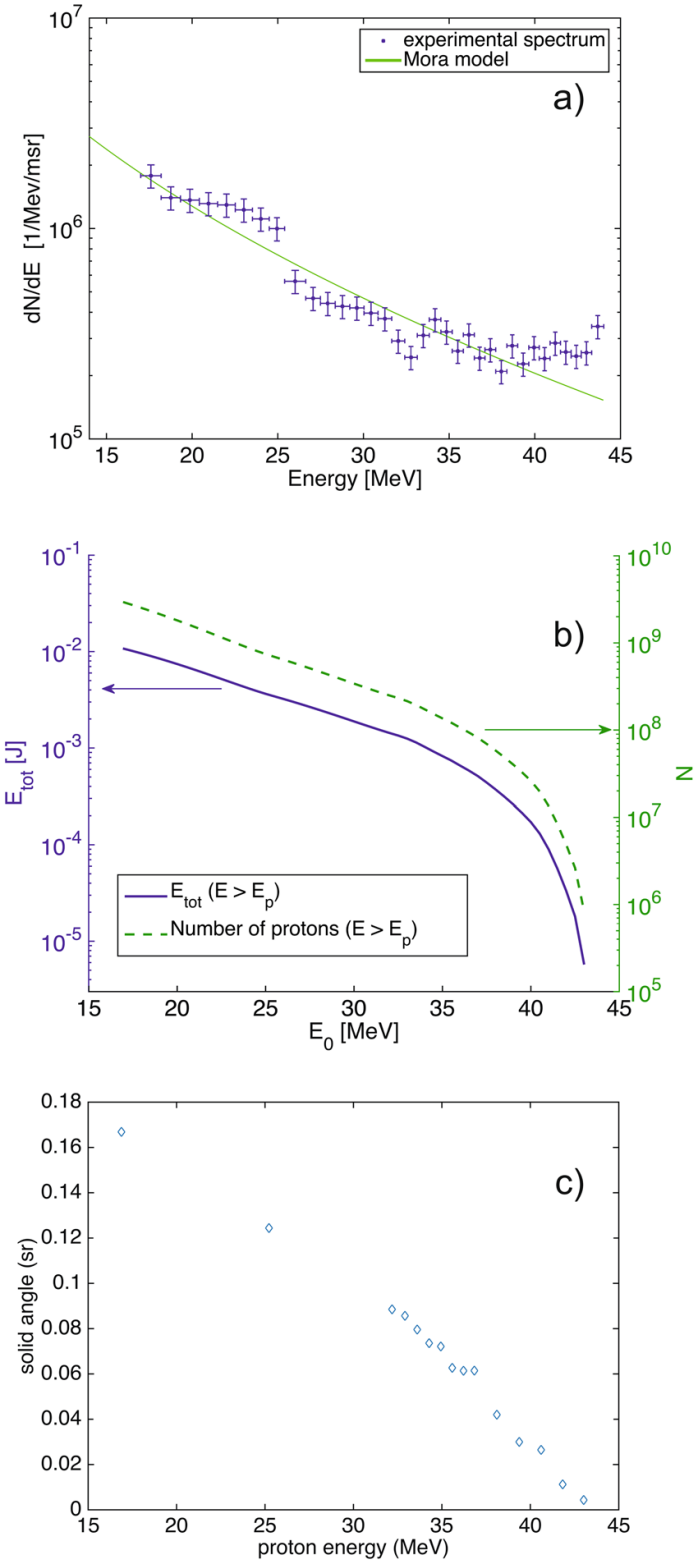
(**a**) Proton energy spectrum retrieved from the RCF stack shown in Fig. 4. (**b**) Total energy E_tot_ contained in the proton beam (solid line) and number of the protons N (dash line) with the energy more than E_0_. (**c**) Variation of the solid angle subtended by the protons as function of their energy.

The angular characteristics^20^ displayed by the proton beam as recorded by the RCF and the monotonous decrease of the proton dose with the energy (see Fig. 6) are both well consistent with the protons being accelerated in the TNSA regime. To retrieve the spectrum shown in Fig. 6a, the radiation dose in each RCF is inferred from the optical density change induced by the deposited proton energy, this according to the absolute calibration of the RCF given in ref.^35^. The energy deposition in each film is calculated using the code SRIM^37^. The solid line represents the fit given by the isothermal plasma expansion model of P. Mora^38^:

$$dN/dE={n}_{e0}{c}_{s}{t}_{acc}{S}_{sheath}/{(2E{T}_{h})}^{1/2}\times \exp (-{(2E/{T}_{h})}^{1/2})$$

where *n*_e0_ is the hot electrons initial electron density, *c*_s_ the sound speed, *t*_acc_ the acceleration time^39^, *S*_sheath_ the electron sheath surface and *E* the proton energy.

The interest of such fitting, as demonstrated by us by comparing parameters retrieved from proton spectra to directly measured electron spectra^40^, is that it allows to reliably retrieve the temperature of the hot electrons generated by the laser and inducing the proton and ion acceleration. Here we retrieve T_h_ = 3.1 ± 0.3 MeV as the hot electron temperature, which is in between the scaling given by refs^41,42^, with the latter yielding much higher temperature since it assumes a large-scale preplasma in front of the target, in which electrons can be heated much more efficiently, which is not the conditions of the present experiment, as discussed above.

The total energy contained in the protons with energy more than E_0_ (depicted on the abscissae) is shown in Fig. 6b as well as the number of accelerated protons.

**Figure 7:**
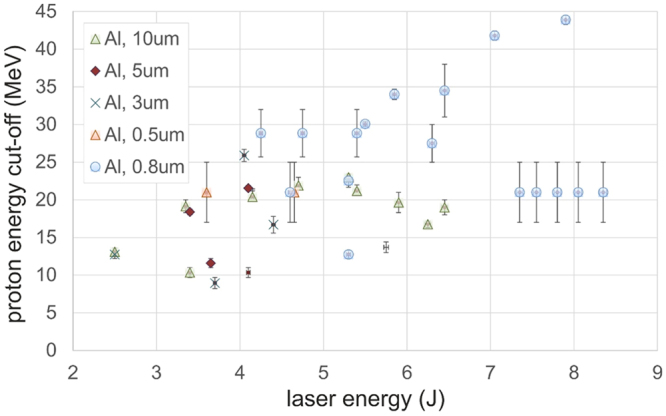
Experimental proton cut-off energy versus laser energy for different target thickness. The difference in the relative error for the proton energy is induced by differences in RCF stack composition used in different shots. The error-bar for the laser energy is 0.5% and is partially hidden by the markers.

In itself, measuring proton beams produced by TNSA does not demonstrate that the rear-surface is gradient-free since TNSA can take place, although in a progressively degraded manner, when there are gradients at the target surface^43^. However, as shown in Fig. 7, we observe that when we reduce the target thickness, the maximum proton energy globally increases, except for some shots that exhibit a sharp drop in energy. This sharp drop exhibited for some shots at the thinnest targets is likely due to the fact that in these shots the targets are indeed affected^39,40^ by the prepulse before the main short pulse. On the other hand, for the shots were we observe a high proton energy that sits on the increasing trend of proton energies, it attests that the rear surface of these targets is in gradient-free conditions. This is certainly so for the shots using 0.8 µm targets and which results are shown in Figs 4–6.

On a side note, we note that the highest measured proton energy is 43.3 MeV, a result which is in line with previous measured obtained at JAEA^45^ also using a high-contrast laser, having similar characteristics, and which constitutes a record for <10 J laser systems.

## Discussion

In the previous Sections, we have assessed the target to be at solid-density with small (front, i.e. based on Fig. 2b, we can assess that around the critical density the plasma gradient exp[−x/L] is characterized by L < 1 µm) and no (rear) gradients, at least at the time of the main laser irradiation and during the few ps over which proton acceleration takes place. Complementarily, the modeling of the X-ray spectra shown in Fig. 3 gives us a measurement of the temperature of the target bulk. The fitting of the continuum, as well as the ratio between the He_α_ and Ly_α_ resonance lines intensities of ~1.4, corresponds well to the emission of a Al plasma at near-solid (~2 × 10^23^ cm^−3^) density and 280–320 eV electron temperature. This provides us with the estimation of the bulk plasma parameters at the front surface of the target in the central heated zone of the target (from which the recombination continuum and resonance lines are mainly emitted) of ~10 µm diameter around the laser focal spot. The latter diameter estimate is based on the fact that if that zone would be wider, then it would fully absorb the emitted keV energy X-rays and would not allow the observed hollow atom states to be pumped; hence we can estimate that beyond that central zone the plasma temperature drops to a temperature of tens of eV.

As we will show now, this characterization of such heating of the target bulk corresponds quite well to what we can model using as an input the parameters of the hot electrons accelerated in the focal spot by the laser at the target front. For this, the damping of the hot electrons energy is evaluated using a simple 1D three-temperature code^46^ that calculates the temperature of the hot electrons T_h_, of the cold (bulk) electrons T_cold_, and of the bulk ions T_i_, and takes into account the expansion of the target. The hot electrons, which are assumed to have a Maxwellian energy distribution, lose energy over time due to (i) collisions with cold electrons and (ii) the development of the resistive electric field within the target that is at the source of the return current.

The initial density of the hot electron injected into the target n_h_ that is used as input in the model can be estimated using an energy balance equation between the absorbed fraction (f) of the laser power and the power that is developed by the hot electrons: f × I_l_ ~ n_h_v_h_T_h_. To estimate the absorption, we run additional PIC simulations (in 1D) using the preplasma profile shown in Fig. 2b irradiated by the main laser pulse. We measure 87% laser absorption in this case (as opposed to only 3% when using a sharp plasma boundary), which shows that high absorption is consistent with the small front gradients discussed above. We note that such high absorption is consistent with the scaling of ref.^47^. In the following, we thus use f = 0.8 as a lower limit for the absorption, a laser intensity I_l_ = 3 × 10^20^ W/cm^2^, and an average value T_h_ = 3.1 MeV as retrieved from the experimental proton spectra, and we obtain n_h_ = 1.6 × 10^22^ cm^−3^. Note that regarding the confidence we can have on the retrieval of the hot electron temperature from the fitting of the spectrum of the protons that are collected at large distances from the target, we have shown that indeed such diagnostic was providing reliable measurements compared to local measurements of the hot electrons when sampled at the target surface^40^. Such a high density, higher than the relativistically enhanced critical density at the laser wavelength (γ_h_n_c_ = 1.2 × 10^21 ^cm^−3^ with γ_h_ = 7.3 in our conditions of T_h_ = 3.1 MeV) is justified as the laser-target interaction is performed in high-contrast conditions, hence the gradient at the target front is sharp, and electrons can be accelerated at high density over the skin depth of the laser penetration into the target.

**Figure 8:**
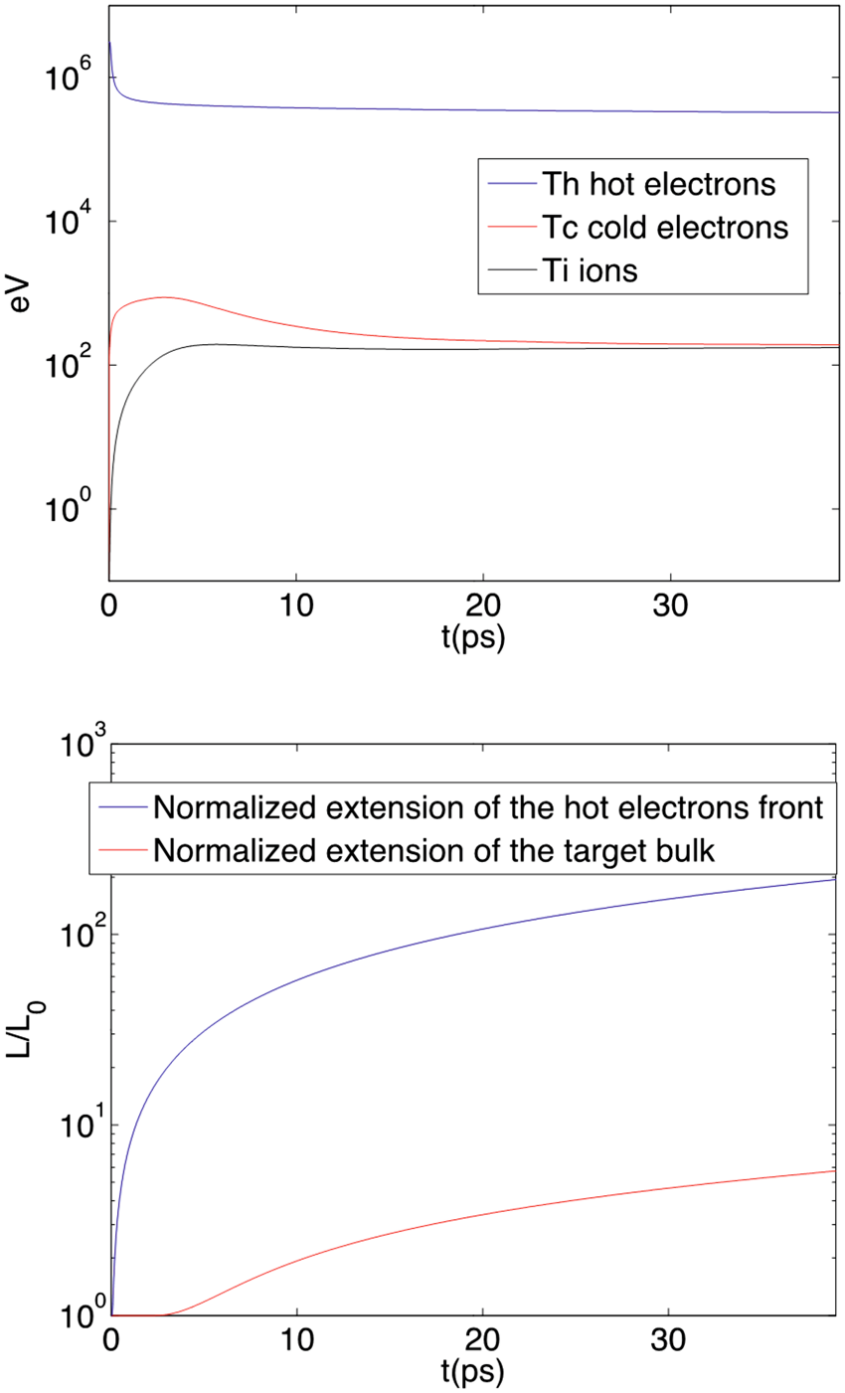
Modeled temporal evolution of (top) T_h_ (the hot electron temperature), T_c_ (the cold electron temperature), and T_i_ (the ion temperature) within a 0.8 µm thick Al target irradiated by a laser having an intensity of 3  × 10^20^ W/cm^2^ and a pulse duration of 60 fs, and (bottom) the normalized extensions (in the 1D model) of the hot electrons front and the target bulk.

Figure 8 (top) illustrates the temporal evolution of T_h_, T_c_ and T_i_ in the Al target. It suggests that the heating of the bulk electrons and ions takes place quite rapidly: already over 10 ps both are heated to ~200 eV and equilibrate over ~20 ps, although the full thermalization of the hot electrons (i.e., when T_h_ = T_c_ = T_i_) takes place on a much longer time-scale. Figure 8 (bottom) illustrates the target expansion, as well as the progression of the hot electrons front as they drive the ion acceleration in the sheath. This further suggests that over the time-scales of the bulk heating mentioned above, the bulk expands little, by a factor 2 (10 ps) or 3 (20 ps), hence remaining at high density.

Obviously, to arrive to a better picture of the target heating, a better model would be required, i.e. one that takes into account, in 3D, the self-consistent propagation of the electrons through the target, in the self-generated magnetic fields^48^, the growth of which is influenced^49^ by the resistivity change the target material is undergoing as fs-timescales. Such improved modelling would ideally integrate atomic physics to describe the generation of radiation and its transport in matter, which impacts its ionization and thus its resistivity^1^. Such improved modelling is actively undertaken by several groups^1,50,51^, but still out of reach and will still require significant effort. The data and simple modelling presented here is intended as a step in this direction by providing better characterization of the heating and of the resulting target state.

## Conclusion

In summary, using two complementary diagnostics, we have been able to characterize the interaction of an ultra-high intensity, and ultra-high contrast laser with a thin Al solid slab. We stress that although the conditions realized here are not gradient-free, as achievable using FELs, we have nonetheless shown that at the time of the main laser pulse interaction, the target front and rear surfaces have small gradients, i.e. at least comparable to the best reported ones so far for high-intensity laser-solid interactions^33^, which were also obtained using an OPCPA laser system. This warrants very good temporal contrast for the laser pulse and using thin targets without having to resort to plasma mirrors^13^ and the associated laser energy loss^52,53^. The fact that there is a non-null front gradient has the benefit that a very efficient coupling between the laser energy and the target is possible (without any gradient, such coupling would be reduced)^54^, and the generation of a very dense population of hot electrons at high temperature (as diagnosed by the accelerated protons). These couple very efficiently to the bulk electrons and ions, heating those (as diagnosed by X-ray spectrometry) to ~300 eV, which is in good agreement with a 1D modeling of such coupling using the hot electrons parameters as an input.

Such efficient heating to high temperatures opens possibilities for probing various materials, using a much smaller-scale and relatively low-cost facility compared to a FEL. We note that for such mid-scale laser, the employed technology is key to allow high-contrast interactions without the need to resort to contrast enhancement techniques. We also note that heating takes place here through the mediation of hot electrons which effect can be undesired for some applications, and also generate secondary x-rays, gamma-rays and particles, of which FEL heating of target is mostly free. Hence, each provide different heating characteristics which need to be considered in view of the desired application. No doubt that the upcoming next generation of multi-PetaWatt (PW) lasers like the future Extreme-Light-Infrastructure (ELI) facilities^55^ will provide even more varied possibilities and extended heating capabilities of solids or even compressed matter.
